# INSCYD physiological performance software is valid to determine the maximal lactate steady state in male and female cyclists

**DOI:** 10.3389/fspor.2024.1376876

**Published:** 2024-05-07

**Authors:** Chiel Poffé, Kaat Van Dael, Reinout Van Schuylenbergh

**Affiliations:** ^1^ Exercise Physiology Research Group, Department of Movement Sciences, KU Leuven, Leuven, Belgium; ^2^ INSCYD GmbH, Salenstein, Switzerland

**Keywords:** maximal lactate steady state, mathematical modeling, VLa_max_, bicycle exercise, INSCYD

## Abstract

**Introduction:**

The maximal lactate steady state (MLSS) is defined as the highest workload that can be maintained without blood lactate accumulation over time. The power output at MLSS (PMLSS) is regularly implemented to define training zones, quantify training progress, or predict race performance. The gold standard methodology for MLSS determination requires two to five trials of constant-load exercise, which limits the practical application in training. The INSCYD software can calculate the PMLSS (PMLSS_INSCYD_) based on physiological data that can be obtained during a ∼1 h laboratory visit. However, to the best of our knowledge, the validity of the most recent software version has not yet been investigated. This study aimed to assess the validity of the software's calculations on PMLSS in cycling.

**Methods:**

The data for this study were retrieved from two published scientific sources. Thirty-one cyclists (19 males, 12 females) performed a 15 s sprint to estimate the VLa_max_, a ramp test for the $\dot{V}O_{2\max}$ assessment, and two to five constant-load tests to determine the PMLSS. The INSCYD software was used to calculate the PMLSS based on the $\dot{V}O_{2\max}$, VLa_max_, sex, body mass, and body composition.

**Results:**

The PMLSS_INSCYD_ was higher than the PMLSS in the entire sample (mean difference: 4.6 W, *p* < 0.05, 95% CI 0.8–8.3 W) and in men (mean difference: 6.6 W, *p* < 0.05, 95% CI 1.3–11.8 W), but not in women (mean difference: 0.8 W, n.s., 95% CI −3.7 to 5.3 W), which was within the typical error of the PMLSS estimations (∼3%). In 12 subjects (nine males, three females), the PMLSS_INSCYD_ differed by 3.1–7.3% compared to the MLSS. The Pearson correlations between the measured PMLSS and the calculated PMLSS (PMLSS_INSCYD_) were very strong in men (*r* = 0.974, *p* < 0.001, 95% CI 0.933–0.99), women (*r* = 0.984, *p* < 0.001, 95% CI 0.931–0.996), and for the entire sample (*r* = 0.992, *p* < 0.001, 95% CI 0.982–0.996).

**Discussion:**

In conclusion, the PMLSS can be accurately calculated using the INSCYD software, but it still requires advanced testing equipment to collect valid $\dot{V}O_{2\max}$ and VLa_max_ data.

## 1 Introduction

The aim of sports science research and practice is to investigate the relationship between an athlete’s physiological response to maximal and submaximal exercise and endurance performance (1). This information can also be used to determine threshold values to demarcate training intensity zones (2), monitor training effects (3), or predict performance (1). Incremental tests are often used to determine the maximal oxygen consumption ($\dot{V}O_{2\max}$) and the lactate threshold, which aim to approximate the maximal lactate steady state (MLSS) (4). The MLSS is defined as the highest workload that can be maintained without blood lactate accumulation over time (5, 6). The MLSS appears to be a valid marker for demarcating heavy from severe exercise domain (2) and predicting endurance performance in time trial cycling (7) or triathlon (8). The determination of the power output at the MLSS (PMLSS) requires two to five constant-load tests at a relatively high intensity within a 1- to 2-week period (5, 6). The PMLSS is defined as the highest workload corresponding to a maximal blood lactate accumulation of 0.02 mmol·L^−1^·min^−1^ in the final 10 min or 0.05 mmol·L^−1^·min^−1^ in the final 20 min of a 20–30 min constant-load effort (5). To avoid such an extensive procedure, which is also tricky to fit into an athlete's schedule, the so-called “anaerobic-threshold” methods based on blood lactate profiles have been developed to predict the PMLSS from a single exercise test. The validity of these threshold methods depends on the test protocol used. In the literature, the duration of the incremental workload steps varies between 3 and 8 min, while the magnitude of the workload steps varies between 10 and 50 W (9, 10). Thus, depending on the applied test protocol, a given threshold method may either be valid or invalid for predicting the PMLSS (4, 11). Furthermore, graded exercise testing that is needed to establish these blood lactate profiles compromises the accurate assessment of the maximal oxygen consumption ($\dot{V}O_{2\max}$), for which a total test duration of a maximum of 8–12 min is advised (12). This means that graded exercise testing does not provide practitioners with a practical solution to accurately assess both the $\dot{V}O_{2\max}$ and the PMLSS in a single-test occasion.

In the 1980s, Mader developed a theoretical mathematical model that allowed the assessment of the PMLSS as the highest power at which the lactate formation rate and the lactate combustion rate reached an equilibrium (13). However, to perform the rather complex calculation of the PMLSS, various input data and variables need to be used, including the $\dot{V}O_{2\max}$ and the maximal lactate formation rate (VLa_max_) of an athlete. Measuring both variables can be completed during one ∼1 h laboratory visit. Nevertheless, calculating the PMLSS from these data requires complex mathematical equations and is, therefore, not practical for coaches (14).

The INSCYD physiological performance software was recently introduced to the market as a practical tool for physiological performance assessment. The software can calculate the lactate formation rate and the lactate combustion rate as a function of exercise intensity and subsequently derive the PMLSS from this analysis. This can be done with a combination of a few submaximal and maximal efforts with lactate monitoring or with direct measurements of the athlete's $\dot{V}O_{2\max}$ and VLa_max_ values. The validity of this application when used with a combination of a few submaximal efforts and one maximal effort has been investigated in male cyclists. Consequently, high levels of agreement were found between the measured and calculated PMLSS and $\dot{V}O_{2\max}$ values (15). On average, the calculated and measured PMLSS values differed by only 2 W (95% CI: −6 to +9 W) with a typical error of 8 W, which is well within the expected day-to-day variability (∼3%) in the PMLSS (16). Since the publication of this study (15), the software has already received an update allowing more flexibility in testing protocols. The validity of the new algorithms in calculating the PMLSS has not yet been published in the scientific literature. The performance of the first software version cannot be generalized to the updated software version; hence, the validity of the new version has yet to be demonstrated. It also remains to be determined whether or not the software can precisely calculate the PMLSS in female subjects because only male subjects were tested in the study of Podlogar et al. (15).

The objective of the current study is to assess the validity of the INSCYD physiological performance software (version 2.0) for the PMLSS calculation based on the $\dot{V}O_{2\max}$ and VLa_max_ derived from golden standard test procedures in male and female cyclists.

## 2 Materials and methods

### 2.1 Subjects

A dataset of 31 cyclists (recreational, *n* = 17; amateur, *n* = 10; professional, *n* = 4) was retrieved from two publications on cycling performance (17, 18) (Table 1). Two female subjects did not complete the $\dot{V}O_{2\max}$ test and the MLSS trials and were excluded from the data analysis. As such, all data analyses were performed on 29 individuals. No additional experiments were performed to increase the sample size because our laboratory was not equipped with the same testing instruments. Using different test equipment would have introduced new sources of error. The study was approved by the Ethics Committee Research UZ/KU Leuven (registration: S68352).

**Table 1:** Subjects’ characteristics.

	Women	Men	Total group
Number	*n* = 10	*n* = 19	*n* = 29
Age (years)	25.3 ± 2.5	28.3 ± 4.4	27.2 ± 4.1
Height (cm)	169 ± 6.5	180 ± 5.7	176 ± 7.9
Body mass (kg)	60.7 ± 5.6	76.0 ± 7.0	70.7 ± 9.8

Mean ± SD.

The methodologies of the testing procedures in both datasets used here were very similar and summarized in the subsequent paragraphs.

In both studies, the subjects were instructed to refrain from strenuous physical activity 24 h before the exercise tests were performed and to adhere to a carbohydrate-rich diet. This procedure was set to prevent premature fatigue caused by glycogen depletion or hypoglycemia, which could affect the test results (19).

All subjects visited the laboratory three to four times to perform bicycle exercise tests. All exercise tests were interspersed with 24–48 h of rest or low-intensity training to allow complete recovery. The exercise tests were performed on a bicycle ergometer (four-strain gauge version, SRM, Jülich, Germany), which was calibrated according to the manufacturer's guidelines. The personal cycling position was set on the ergometer, and the subjects used their personal cycling shoe-pedal system. The seat height, seat length, and saddle slope were recorded and used for all the cycling tests. The cycling data were captured by the ergometer display (SRM PowerControl III) and stored at 0.1 s (sprint test) or 1 s (all other tests) intervals and transferred to a personal computer for further analysis.

### 2.2 Anthropometric measurements

The first visit involved the collection of anthropometric and personal information [i.e., age, height, and body mass (Seca, Hamburg, Germany] and body composition (Biamed, BiaMedizintechnik, Köln, Germany). No body composition data were available for the female subjects. The INSCYD's algorithms use body fat percentage to calculate the PMLSS_INSCYD_; thus, a fixed body fat percentage of 20% was used. This value was chosen based on the scientific data of female cyclists with similar body mass and size (20). The individual fat percentage may differ from the fixed value; therefore, we calculated this effect on the PMLSS_INSCYD_.

### 2.3 VLa_max_ test

A 15 s sprint test was performed according to the literature to test the maximum lactate formation rate (17). The subjects performed a 12 min warm-up on the bicycle ergometer at 1.5 W·kg^−1^ [study of Kleinschmidt (18)] or 2 W·kg^−1^ [study of Weber (17)]. This workload corresponded to the easy/moderate intensity domain. The subjects were free to use their preferred cadence to minimize peripheral fatigue (21). After the warm-up, the subjects rested for 10 min by sitting on a chair. This procedure was implemented to minimize the impact of oxidative metabolism on the subsequent sprint. Meanwhile, three capillary blood lactate samples (20 µl) were taken from the earlobe to determine the resting blood lactate concentration (BLC_pre_) (EBIO Plus, Eppendorf, Hamburg, Germany). Thereafter, the subjects performed a 15 s all-out sprint on the bicycle ergometer (SRM, Germany). The kinetic energy of the ergometer's flywheel can be adjusted by changing the transmission ratio. The transmission ratio on the ergometer was chosen as a function of sex and individual body mass (Table 2). During the 15 s test, the subjects had to sprint from a seated position. The ergometer was set in isokinetic mode at a fixed cadence of 130 revolutions per minute. According to the scientific literature, the cadence associated with the highest power output is approximately 110–150 rev·min^−1^. Highly trained cyclists and elite track sprinters usually have higher optimal cadences (22–24). The subjects were verbally encouraged throughout the test. After the test, the subjects rested for 10 min by sitting still on a chair. The blood samples were collected immediately after the end of the sprint and at 1 min intervals during the next 10 min to determine the maximal lactate concentration (BLC_max_) (EBIO Plus, Eppendorf, Hamburg, Germany).

**Table 2:** Ergometer settings (transmission ratio) for the VLa_max_ test as a function of sex and body mass.

Body mass		Transmission ratio	
>	≤	Female	Male
50	60	39 × 26	–
60	70	39 × 26	39 × 23
70	80	39 × 23	39 × 21
80	90	39 × 19	39 × 19
90	100	39 × 17	39 × 17
100	110	–	39 × 15

The maximum lactate formation rate (VLa_max_) was calculated as the ratio of lactate accumulation over the time of lactate production according to the following Equation (1) (25, 26):

(1) $$\mathrm{VL}a_{\max}=\frac{\mathrm{BL}C_{\max}-\mathrm{BL}C_{\mathrm{pre}}}{15s-t_{\mathrm{alac}}}$$

**Figure 1:**
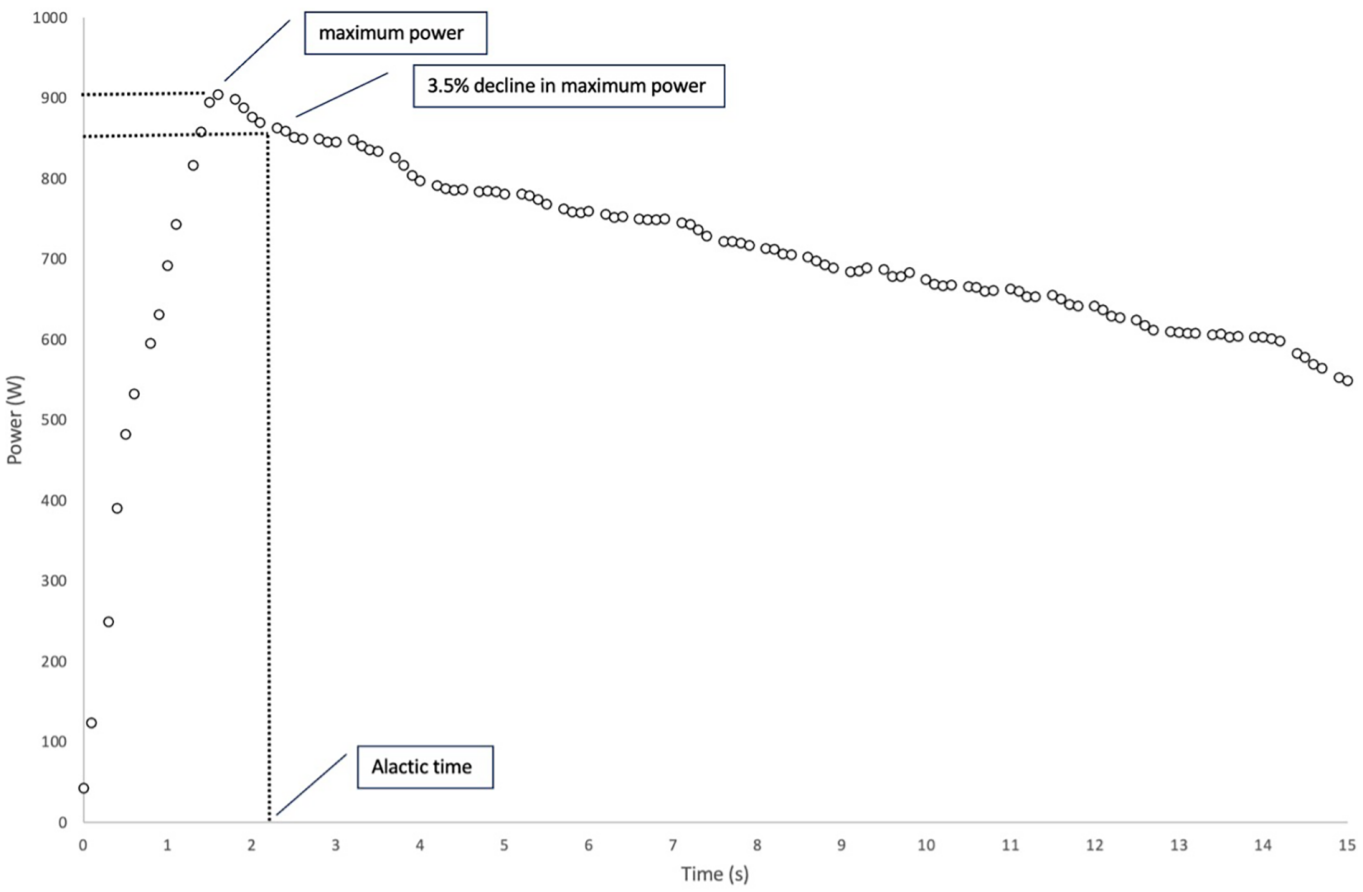
Example of the power output during a 15 s maximal isokinetic sprint. The alactic time (*t_alac_*) is defined as the time when the power output irreversibly decreases by 3.5%.

*t*_alac_ is defined as the period at the beginning of exercise for which (fictitiously) no lactate formation is assumed (26). In this work, *t*_alac_ is determined as the time from the beginning of the sprint (0 s) to when the maximum power decreased irreversibly by 3.5% (Figure 1). The assumption of an irreversible 3.5% power reduction seems favorable because the measurement tolerance of the used SRM system is ∼2.5%. Thus, a power reduction of 3.5% or more cannot be a measurement error of the SRM system but only a real power reduction (17). To make this methodology applicable to all subjects, the ergometer settings were adjusted to the body mass of the athlete separately for males and females to try to account for the different percentages of active muscle mass in both sexes.

### 2.4 $\dot{V}O_{2\max}$ test

A ramp test was performed to assess the maximal oxygen consumption rate ($\dot{V}O_{2\max}$). The subjects cycled for 12 min at 1.5 W·kg^−1^ [study of Kleinschmidt (18)] or 2 W·kg^−1^ [study of Weber (17)] at a freely chosen cadence. After this warm-up, an initial load for the first 2 min was set according to Table 3 depending on the rider class and body mass. This was followed by the ramp test with a load increase of 25 W every 30 s and the ergometer in a speed-independent mode.

**Table 3:** Loads for the first 120 s in the *V*O_2max_ test as a function of sex, rider class, and body mass.

Body mass (kg)		Recreational athletes	Amateur	Professional
>	≤	Power (Watt)		
Female athletes only		100		
60	70	150	180	240
70	80	175	210	280
80	90	200	240	320
90	100	225	270	360
100	110	250	300	400

Breathing gasses were collected breath-by-breath (Oxycon Alpha, Jaeger, Höchberg, Germany). The test was continued until the subjects, despite verbal encouragement, reached physical exhaustion. The test was considered successful if the following criteria were met: (1) a maximum RQ of 1.1 or higher and (2) a plateau in the $\dot{V}O_{2}$ (<150 ml·min^−1^) with a continuously increasing load (27). The $\dot{V}O_{2\max}$ was averaged from the highest 30 s $\dot{V}O_{2}$ values.

### 2.5 Measuring and calculating the MLSS

Based on the measured VLa_max_ and $\dot{V}O_{2\max}$ and the subject's anthropometrics, the PMLSS was calculated using the INSCYD physiological performance software (version 2.0, INSCYD GmbH, Salenstein, Switzerland) (PMLSS_INSCYD_). Individual data used to run these calculations were sex, body mass, body fat %, $\dot{V}O_{2\max}$, and VLa_max_. All other settings in the software, such as detailed body composition and gross efficiency, were kept at default values as a software preset. The software's algorithms normalized the VLa_max_ to the body mass and body composition using a two-compartment model. It then calculated the energy contribution from aerobic and glycolytic energy sources under steady-state conditions. From this, a causational PMLSS is calculated as the highest power output at which the equilibrium of aerobic lactate combustion and glycolytic lactate production is achieved, hence resulting in zero net calculated lactate accumulation. To verify these calculations, we used the PMLSS as provided by Weber (17) and Kleinschmidt (18). In these two publications, the gold standard of the constant-load tests was used, that is, 20 min in Weber's work and 30 min in Kleinschmidt's work. If a steady-state BLC is found (<0.2 mmol·L^−1^ increase in the last 10 min of the test), the load is increased by 10 W at the next constant-load test. If the BLC increases to more than 0.2 mmol·L^−1^, the load is decreased by 10 W during the next constant-load test. The highest workload without a continuous increase (<0.2 mmol·L^−1^) in the BLC in the last 10 min of the constant-load test was considered to represent the PMLSS (5, 28). This criterium was within the accuracy of the lactate analyzer (29).

To assess the robustness of the INSCYD algorithms, we investigated the impact of the typical error and day-to-day variability of the $\dot{V}O_{2\max}$ (±3.3 ml·kg^−1^·min^−1^) and VLa_max_ (±0.11 mmol·L^−1^·s^−1^) measurements on the PMLSS calculation (30). We had no individual data on the body composition of female subjects; hence, the effect of the body fat percentage (±6%) on the PMLSS_INSCYD_ was evaluated.

### 2.6 Statistical analysis

A Shapiro–Wilk test was applied to verify the normal data distribution. All data are expressed as mean ± SD. The Pearson product correlations (*r*) and 95% CI were calculated to quantify the correlation between PMLSS vs. PMLSS_INSCYD_. The Pearson correlation coefficients were quantified as small (*r* < 0.4), moderate (0.40 < *r* < 0.59), and high (*r* > 0.6) (31).

A Bland–Altman plot analysis (expressed as the mean ± 1.96 SD) was used to quantify the bias and the range of agreement between the PMLSS and the PMLSS_INSCYD_ with 95% limits of agreement. If the limits of agreement were less than 3% for the PMLSS, the PMLSS_INSCYD_ was considered in agreement and was, therefore, interchangeable.

The difference between PMLSS vs. PMLSS_INSCYD_ was tested using a Student’s paired *t*-test.

The significance level was set at *p* < 0.05. Statistical analyses were performed using JASP 0.16.04 (https://jasp-stats.org/, Amsterdam, Netherlands) and Microsoft Excel (Microsoft 365, Microsoft Corporation, Redmond, USA).

## 3 Results

The physiological performance characteristics of the subjects are shown in Table 4.

**Table 4:** Subjects’ physiological performance characteristics.

	Women	Men	Total group
Number	10	19	29
$\dot{V}O_{2\max}$ (ml·L^−1^·min^−1^)	49.1 ± 5.0	64.9 ± 7.3	59.4 ± 10.0
*V*La_max_ (mmol·L^−1^·s^−1^)	0.50 ± 0.15	0.59 ± 0.15	0.56 ± 0.15
MLSS BLC (mmol·L^−1^)	4.69 ± 2.04	3.73 ± 0.80	4.06 ± 1.42
PMLSS (W)	179.2 ± 21.9	295.1 ± 44.1	254.8 ± 67.8
PMLSS_INSCYD_ (W)	180 ± 27.9	301.7 ± 47.5	259.7 ± 71.9
PMLSS_INSCYD_ (% $\dot{V}O_{2\max}$)	76.6 ± 5.4	76.7 ± 6.1	76.6 ± 5.8

Mean ± SD.

**Figure 2:**
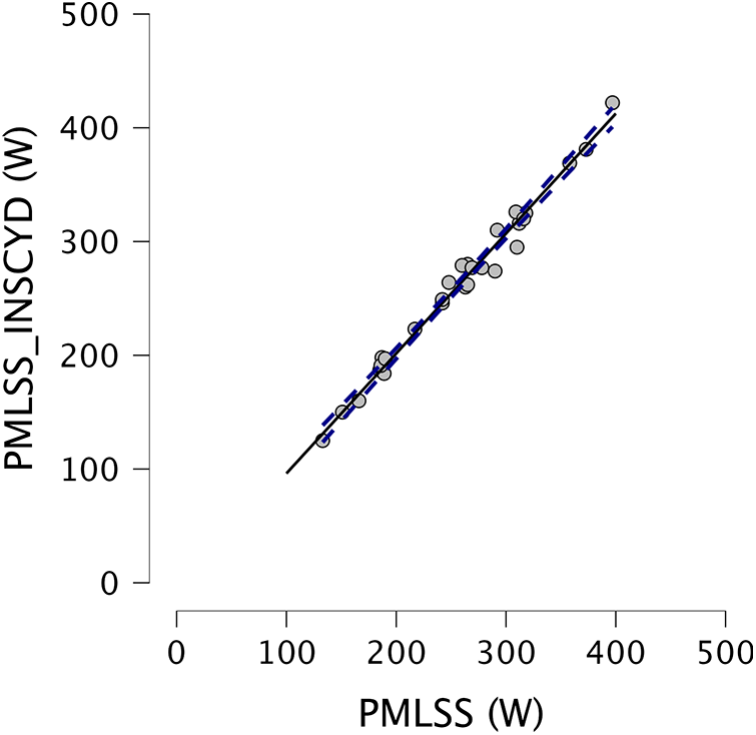
A Bland–Altman plot between the measured PMLSS and the calculated PMLSS (PMLSS_INSCYD_).

The PMLSS_INSCYD_ was higher than the PMLSS in men (mean difference: 6.6 W, *p* < 0.05, 95% CI 1.3–11.8 W, with a range of −16 to 25 W), but not in women (mean difference: 0.8 W, n.s., 95% CI −3.7 to 5.3 W, with a range of −8 to 11 W). For the entire sample, the bias was 4.6 W (*p* < 0.05, 95% CI 0.8–8.3 W, with a range of −16 to 25 W). The Bland–Altman plot between the PMLSS and the PMLSS_INSCYD_ is presented in Figure 2. The BLC at the MLSS averaged 4.06 ± 1.42 mmol·L^−1^ (range, 2.20–5.85 mmol·L^−1^).

**Figure 3:**
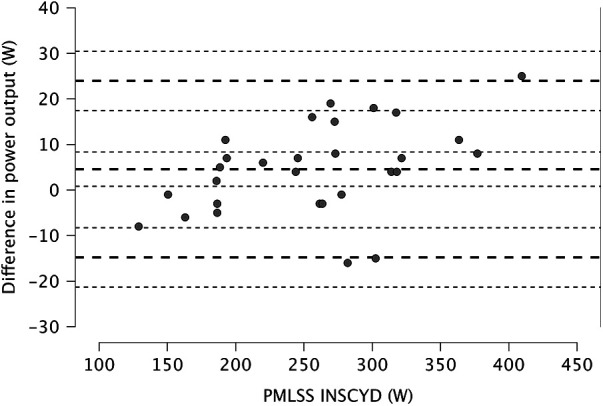
Correlation between the measured PMLSS and the calculated MLSS (PMLSS_INSCYD_). The solid line represents the regression line. The dashed lines represent 95% confidence limits.

The Pearson correlations between the PMLSS and the PMLSS_INSCYD_ were very strong in men (*r* = 0.974, *p* < 0.001, 95% CI 0.933–0.99), women (*r* = 0.984, *p* < 0.001, 95% CI 0.931–0.996), and for the entire sample (*r* = 0.992, *p* < 0,001, 95% CI 0.982–0.996) (Figure 3). The PMLSS can be calculated according to the following Equation (2):

(2) $$\mathrm{PMLSS}=0.933\;*\;\mathrm{PMLS}S_{\mathrm{INSCYD}}+12.85$$

We investigated the impact of the typical errors and the day-to-day variability of the $\dot{V}O_{2\max}$ (±3.3 ml·kg^−1^·min^−1^) and VLa_max_ (±0.11 mmol·L^−1^·s^−1^) measurements on the PMLSS_INSCYD_ (30). Increasing the measured $\dot{V}O_{2\max}$ with 3.3 ml·kg^−1^·min^−1^ increased the PMLSS_INSCYD_ by 19 ± 2 W (6 ± 1% in the male subgroup, 10 ± 2% in the female subgroup). Decreasing the measured $\dot{V}O_{2\max}$ with 3.3 ml·kg^−1^·min^−1^ reduced the PMLSS_INSCYD_ by 17 ± 4 W (7 ± 1% in both sexes). Increasing the VLa_max_ with ±0.11 mmol·L^−1^·s^−1^ decreased the PMLSS_INSCYD_ by 12 ± 4 W (5 ± 1% in the male subgroup, 4 ± 1% in the female subgroup). Decreasing the VLa_max_ with ±0.11 mmol·L^−1^·s^−1^ increased the PMLSS_INSCYD_ by 15 ± 3 W (5 ± 1% in the male subgroup, 8 ± 1% in the female subgroup). Increasing or decreasing the $\dot{V}O_{2\max}$ or VLa_max_ increased the average bias with the measured PMLSS (±13 W) without affecting the correlation (*r* = 0.99, *p* < 0.001).

The effect of error in the body fat percentage on the calculation of the PMLSS_INSCYD_ in the female subjects was also calculated. In the 20 ± 6% range, we found that for a Δ1% in the body fat percentage, the PMLSS_INSCYD_ changes with ∼1 W without affecting the correlation (*r* = 0.98, *p* < 0.001).

## 4 Discussion

### 4.1 Prediction of the MLSS

The main objective of this study was to verify whether the INSCYD physiological performance software can precisely calculate the PMLSS in cycling exercise based on sex, anthropometric data, $\dot{V}O_{2\max}$, and VLa_max_ from a standardized ramp protocol and a 15 s maximal isokinetic sprint. These data can be captured in a ∼1 h single laboratory visit and could be a more practical solution for the PMLSS determination compared to golden standard procedures. In this study, we detected a good agreement between the PMLSS_INSCYD_ and the PMLSS (*Δ*4.6 W). The PMLSS_INSCYD_ was within the typical daily variation of the PMLSS when obtained from the gold standard test protocol and can thus be considered valid. Similar results were found by Podlogar et al. in 11 endurance-trained male cyclists ($\dot{V}O_{2\max}$ 61.0 ± 7.9 ml·kg^−1^·min^−1^) (15). They observed a typical error for the PMLSS_INSCYD_ of 8 W, which was smaller than the 3% typical day-to-day variability of PMLSS values (16). It should be noted that in the study of Podlogar et al., $\dot{V}O_{2\max}$ and VLa_max_ were calculated from the lactate and power data of six intervals performed at various intensities and durations. Otherwise stated, they used the INSCYD physiological performance software differently with data sources that have different typical errors and different errors in the calculated PMLSS. Consequently, it is of utmost importance to use high-quality equipment and sampling techniques to minimize the error in the input data and improve the calculation accuracy. The Monte Carlo analysis can provide insight into the error of the calculated result based on the typical errors of the input data (32).

In our study, the PMLSS was assessed by applying 10 W increments in subsequent constant-load trials. Consequently, the measured PMLSS had an average accuracy level of ±5 W (33). Interestingly, the bias between the calculated and measured PMLSS (∼5 W) values corresponded well with the accuracy level of the applied PMLSS methodology. In the study of Podlogar et al., no significant bias was observed between the measured PMLSS and that derived from the INSCYD calculations (Δ2 W, n.s.) (15). They used 5 W increments for the PMLSS testing, thereby obtaining an average accuracy that was ±2.5 W (33). It can be speculated that with smaller workload increments in our PMLSS methodology, the bias with MLSS_INSCYD_ would have been reduced. This needs to be verified in future research. Despite this methodological limitation, the bias observed in our study was still lower than the ∼3% typical day-to-day variation that can be expected from PMLSS values (16). It should be noted that in nine male subjects and three female subjects, the bias between PMLSS and PMLSS_INSCYD_ exceeded 3% (range, 3.1–7.3%). These differences in the calculated PMLSS, when compared to the measured PMLSS, were within the range of the calculated bias caused by the day-to-day variability in the $\dot{V}O_{2\max}$ (±3.3 ml·kg^−1^·min^−1^) and VLa_max_ (±0.11 mmol·L^−1^·s^−1^) measurements. These inaccuracies in the PMLSS_INSCYD_ can be attributed to the inaccuracies of the input data and not necessarily to inaccurate algorithms.

### 4.2 Limitations

Our study provides quantitative information to evaluate the accuracy of the INSCYD software in calculating the PMLSS. However, some limitations of this study should be acknowledged. To feed the algorithm of the software, measurements of age, body mass, body fat %, $\dot{V}O_{2\max}$, and VLa_max_ are required. In our study, $\dot{V}O_{2\max}$ and VLa_max_ were measured using golden standard procedures with lab-accurate equipment ensuring optimal data quality. The ramp protocol was adjusted based on sex, body mass, and rider class. This procedure was implemented to ensure exhaustion times of 8–12 min, as generally recommended for $\dot{V}O_{2\max}$ testing (12). It should be noted that the availability of high-end testing equipment might be limited in practice; hence, the accuracy of PMLSS_INSCYD_ can be affected by equipment of a lower-quality standard.

Unfortunately, the level of data quality obtained for $\dot{V}O_{2\max}$ and VLa_max_ was not available for the body fat assessments. A bio-electrical impedance method was used for the male subjects. This technology is often used in a practical setting because of its low price and user-friendly operation. It uses the electrical properties of the body to estimate the total body water, from which the body fat mass is estimated (34). The potential error sources are variations in limb length, recent physical activity, nutrition and hydration status, tissue temperature, blood chemistry, ovulation, and electrode placement. According to the literature, bio-electric impedance methods underestimate the body fat composition in a normal-weight population in comparison with a reference method (35). Therefore, the body fat percentage derived from bio-electric impedance technologies should be interpreted with caution. No body fat measurements were available from the female subjects. Therefore, the body fat was estimated by a fixed reference value in female cyclists with similar body mass and size. Despite these methodological limitations in body fat assessment, the effect on PMLSS_INSCYD_ is relatively small because a 1% difference in body fat results in ΔPMLSS_INSCYD_ ∼1 W.

The current validation study was performed on 29 subjects. Although it is not uncommon in similar validation studies to use relatively small sample sizes, future research should preferably investigate larger sample sizes to increase the statistical power.

### 4.3 Practical applications

The PMLSS attracts the interest of exercise physiologists and practitioners in prescribing training zones for endurance exercises (2) and monitoring training effects (3) and as a performance prediction metric (7, 8). While golden standard MLSS protocols require typically two to five exercise tests, the data needed to feed the INSCYD algorithms can be captured in a single-test occasion of ∼1 h. Therefore, this is likely to be a more practical and time-efficient approach in an athlete population. Different from the lactate-threshold concepts, this novel calculation method is based on $\dot{V}O_{2\max}$ and VLa_max_. Hence, in contrast to conventional lactate profile testing using incremental loads, this approach does not only provide an accurate PMLSS but also a separate analysis of the maximum aerobic and anaerobic markers and a more complete picture of an athlete's physiology. This knowledge can aid coaches when prescribing more effective training recommendations.

To the best of our knowledge, the current study is the second study to test the validity of the INSCYD physiological performance software in calculating the PMLSS in cycling exercise and the first to include female subjects. The utility of the INSCYD software in non-athletic populations with typically lower cardiorespiratory fitness parameters ($\dot{V}O_{2\max}$), such as in cardiac patients, must still be explored.

## 5 Conclusions

The data in this study demonstrate that the INSCYD physiological performance software can accurately calculate the PMLSS based on the data from a *V*O_2max_ ramp protocol and a 15 s maximal isokinetic sprint in both male and female subjects. The INSCYD physiological performance software appears to be a valid and practical tool for assessing the PMLSS in a male and female endurance-trained population. However, to feed the software with valid $\dot{V}O_{2\max}$ and VLa_max_ values, advanced testing equipment, such as a metabolic cart, a lactate analyzer, and an accurate ergometer, are still needed.

## Data Availability

The original contributions presented in the study are included in the article/supplementary material, further inquiries can be directed to the corresponding author/s.
